# Deaths from Anæsthetics

**Published:** 1898-02-05

**Authors:** 


### DEATHS FROM ANÆSTHETICS.

(*Continued from page* 310.)

The following is another instalment of the list of deaths from anæsthetics during the past year which we have collected. We hope to complete the list next week:—

The *Evening News*, September 8th, reports an inquest on a boy, aged eight years, who died under chloroform at the Plaistow Hospital.

The *Morning Advertiser*, September 15th, reports an inquest on a man, aged 37, who died in University College Hospital while under the influence of ether which had been administered for an operation for strangulated hernia.

The *Western Daily Press*, September 15th, reports an inquest on a woman, aged 65, who died at the Bristol General Hospital while ether was being administered. The case was one of intestinal obstruction, the patient was in a very bad condition, and death took place before she was fully under.

The *Daily Mail*, September 17th, reports an inquest on a woman who died at Oxford while under ether, administered for the purpose of an operation being performed.

The *Longton Times*, September 17th, reports an inquest on a youth, aged 17, who died while under chloroform at the Cottage Hospital, Barton-under-Needwood. The operation was for a diseased gland in the neck.

The *Nottingham Guardian*, October 2nd, reports an inquest on a child (A. E. Shlipston), age 5½ years, who died under chloroform administered for the opening of an abscess in the neck, which was rapidly spreading and affected the trachea.

The *Evening Standard*, October 4th, reports an inquest concerning the death of a man, age 52, who expired suddenly on September 30th, at University College Hospital, after the administration of an anæsthetic. Chloroform had been administered preparatory to an operation for "cancer of the face." The patient struggled a little at first, and the anæsthetic was withheld for a few moments. He was then placed on the operating table, when his colour changed, and he expired almost immediately from syncope.

The *Yorkshire Herald*, October 5th, reports an inquest on a man, aged 36, who died at the Stockton Hospital while under chloroform, which was being administered for an operation for fistula. He resisted the action of the chloroform, and died before the operation was commenced.

The *Southampton Echo*, October 6th, reports an inquest on a man, aged 44, who died at the Royal South Hants Infirmary. He suffered from peritonitis. Ether was given, but just as the operation was about to be commenced the patient vomited badly, and the anæsthetic was changed, chloroform being administered. Syncope occurred, and the patient died.

The *Liverpool Post*, October 7th, reports an inquest on a man, aged 69, who died in the Toxteth Workhouse while chloroform was being administered. He was a bad subject, had drunk a good deal, suffered from chronic bronchitis, and had a carbuncle on his neck, for which an operation was about to be performed. He took the chloroform well, but just as the operation was about to be commenced he collapsed.

